# Saccharides Influence Sperm Quality and Expressions of Motility and Fertilization-Associated Genes in Cryopreserved Sperm of Pacific Abalone, *Haliotis discus hannai*

**DOI:** 10.3389/fcell.2022.935667

**Published:** 2022-07-19

**Authors:** Shaharior Hossen, Zahid Parvez Sukhan, Yusin Cho, Cheol Young Choi, Kang Hee Kho

**Affiliations:** ^1^ Department of Fisheries Science, College of Fisheries and Ocean Sciences, Chonnam National University, Yeosu, South Korea; ^2^ Division of Marine BioScience, National Korea Maritime and Ocean University, Busan, South Korea

**Keywords:** sperm cryopreservation, saccharides, mRNA expression, ATP content, fertilization, Pacific abalone

## Abstract

Pacific abalone, *Haliotis discus hannai*, is a highly commercial seafood in Southeast Asia. The present study aimed to determine the influence of saccharides and vitamins on post-thaw sperm quality, ATP content, fertilization capacity, hatching capacity, and mRNA content of motility and fertilization-associated genes of Pacific abalone. Sperm cryopreserved using saccharides improved the post-thaw sperm quality including motility, acrosome integrity (AI), plasma membrane integrity (PMI), and mitochondrial membrane potential (MMP). However, vitamins (l-ascorbic acid) did not result in any significant improvement in sperm quality. Sperm cryopreserved using saccharides also improved ATP content, DNA integrity, and mRNA content of motility and fertilization-associated genes of post-thaw sperm than sperm cryopreserved without saccharides. Among sperm cryopreserved using different saccharides, post-thaw sperm quality indicators (except PMI) and mRNA content of motility and fertilization-associated genes did not show significant differences between sperm cryopreserved using 3% sucrose (S) combined with 8% dimethyl sulfoxide (DMSO) and sperm cryopreserved using 1% glucose (G) combined with 8% ethylene glycol (EG). However, sperm cryopreserved using 3% S + 8% DMSO showed higher post-thaw sperm quality (motility: 58.4 ± 2.9%, AI: 57.1 ± 3.2%, PMI: 65.3 ± 3.3%, and MMP: 59.1 ± 3.2%), ATP content (48.4 ± 1.8 nmol/ml), and % DNA in tail (2.09 ± 0.20%) than sperm cryopreserved using other saccharides. When sperms were cryopreserved using 3% S + 8% DMSO, the mRNA content of motility (heat shock protein 70, HSP70; heat shock protein 90, HSP90; protein kinase A, PKA-C; axonemal protein 66.0, Axpp66.0; and tektin-4) and fertilization-associated (sperm protein 18 kDa, SP18 kDa) genes were higher than in sperm cryopreserved using other saccharides. However, changes in the mRNA contents of these genes were insignificant between sperm cryopreserved using 3% S + 8% DMSO and 1% G + 8% EG. Taken together, these results indicate that cryopreservation using 3% S + 8% DMSO can improve post-thaw sperm quality and mRNA contents better than other examined cryoprotectants. The present study suggests that 3% S + 8% DMSO is a suitable cryoprotectant for sperm cryopreservation and molecular conservation of this valuable species.

## 1 Introduction

Pacific abalone, *Haliotis discus hannai* is high-priced seafood highly demandable in Korea and Southeast Asia (Sukhan et al., 2022). It contains bioactive molecules with antioxidant and anticancer activities that are beneficial for human health (Suleria et al., 2017; Hossen et al., 2021a). Among invertebrate species, the Pacific abalone is the main commercial aquaculture species in Korea (Sharker et al., 2020; Sukhan et al., 2021). Commercial aquaculture of abalone mostly depends on hatchery-produced seeds (Sukhan et al., 2022). Seed production of Pacific abalone using *in vitro* fertilization requires good quality and a high quantity of sperm (Kim et al., 2020a). Cryopreserved sperm can solve those issues by supplying sperm through *in vitro* fertilization (Liu et al., 2014a; Kim et al., 2020a). Cryopreservation is an auspicious biotechnique that can ensure the conservation of genetic diversity and a continuous supply of sperm (Gheller et al., 2019; Kim et al., 2020b). Sperm cryopreservation is widely applied to provide gametes throughout the year for artificial insemination or to ensure alternative broodstock management (Iorio et al., 2019; Hossen et al., 2021c).

The success of cryopreservation mostly depends on the types and concentrations of cryoprotectants (CPAs) (Soni et al., 2019; Hossen et al., 2021c). CPAs are categorized into basic two types (penetrating CPA and non-penetrating CPA) depending on their cell membrane permeability (Jang et al., 2017). Penetrating CPAs (P-CPAs) reduce ice growth and cell dehydration during cryopreservation (Wowk, 2007). Non-penetrating CPAs such as saccharides, protein, amino acids, and vitamins are known to play protective roles against extracellular ice crystallization (Cabrita et al., 2011; Zhu et al., 2017; Ariyan et al., 2021). Non-penetrating CPAs such as saccharides or vitamins combined with penetrating CPAs have been recently used to improve the quality of post-thaw sperm of fish and shellfish (Liu et al., 2014a; Liu et al., 2014b; Liu et al., 2016; Sandoval-Vargas et al., 2021). Saccharides such as glucose and sucrose can stabilize phospholipids in the cell membrane and decrease the toxicity of CPAs during cryopreservation (Hossen et al., 2021c). A vitamin (ascorbic acid) is a water-soluble antioxidant which can break chain reactions and remove free radicals during cryopreservation (Zhang et al., 2012). Ascorbic acid can act as a free radical scavenger by producing monodehydroascorbate radicals that can prevent oxygen or other molecules from producing more reactive radicals (Alamaary et al., 2020).

Despite its benefit, cryopreservation can reduce the intracellular adenosine triphosphate (ATP) content of sperm (Kommisrud et al., 2020). ATP is the main source of biochemical energy that can regulate sperm motility (Cosson, 2012). Cryopreservation is known to decrease the quality of sperm by damaging the acrosomal membrane, plasma membrane, and mitochondrial membrane (Xin et al., 2018; Soni et al., 2019; Hossen et al., 2021c; Khan et al., 2021). Plasma membrane integrity (PMI), mitochondrial membrane potential (MMP), and acrosome integrity (AI) are indicators of the quality of cryopreserved sperm (He and Woods, 2004; Hossen et al., 2021a). The fluorescent technique is an important method to evaluate sperm quality. Recently, this technique has been applied to assess PMI, MMP, and AI of shellfish sperm (Pereira et al., 2010; Liu et al., 2014a; Kim et al., 2020a; Hossen et al., 2021a; Hossen et al., 2021b). Cryopreservation can also denature sperm deoxyribonucleic acid (DNA) integrity which can be used to assess the success of sperm cryopreservation (Hossen et al., 2021c; Khan et al., 2021; Kuo and Gwo, 2022).

Knowledge about the molecular basis of the cryopreservation process and sperm damage is limited. Cryopreservation can alter mRNA stability, gene and protein abundance, and epigenetic content of sperm (Hezavehei et al., 2018). Cryopreservation using only penetrating cryoprotectants (P-CPAs) can significantly reduce the mRNA abundance of heat shock proteins (HSP70 and HSP90) in oyster and Pacific abalone sperm (Riesco et al., 2019; Hossen et al., 2021a). HSP70 could activate Ca_2_^+^-ATPase activity, whereas HSP90 is engaged in intracellular calcium homeostasis and protein tyrosine phosphorylation of sperm (Li et al., 2014; Zhang et al., 2015). Calcium is a crucial secondary messenger that can control sperm motility (Alshawa et al., 2019). Cryopreservation using P-CPAs can also suppress the mRNA abundance of protein kinase A (PKA-C) in Atlantic salmon and Pacific abalone sperm (Lee-Estevez et al., 2019; Hossen et al., 2021a). PKA-C can regulate CatSper channels which is crucial for the motility and fertility of sperm (Mannowetz et al., 2017; Orta et al., 2018). PKA-C can phosphorylate axonemal proteins (Axp). Axp66.0 has two PKA-C phosphorylation sites that might regulate sperm motility (Sukhan et al., 2020; Speer et al., 2021). Tektins are an important family of proteins in the axoneme. They are the main components of the sperm flagellum cytoskeleton that can regulate sperm motility (Cao et al., 2018; Alshawa et al., 2019). Cryopreservation can also suppress tektins in post-thaw sperm of abalone (Sukhan et al., 2022).

To date, information on Pacific abalone sperm cryopreservation is limited. Previously published studies have reported sperm cryopreservation based on intracellular CPAs and antifreeze proteins (Kim et al., 2020a; Hossen et al., 2021a; Hossen et al., 2021b). However, the effects of saccharides and vitamin on the post-thaw sperm quality of Pacific abalone have not been reported yet. It is presently unclear whether supplementation of saccharides or vitamins as non-penetrating CPAs with P-CPAs could improve the post-thaw sperm quality of Pacific abalone. There are limited studies on gene expression fluctuations of important regulatory genes involved in motility and their relationship with the fertilizing potential of cryopreserved Pacific abalone sperm. Hence, the present study aimed to assess the effects of supplementation of different saccharides or vitamins with P-CPAs on the post-thaw sperm quality of Pacific abalone. Cryopreserved sperm quality was determined based on motility, AI, PMI, MMP, DNA integrity, ATP content, and fertilization capacity. qRT-PCR was performed to quantify the mRNA content of sperm motility-associated genes (HSP70, HSP90, PKA-C, Axp 66.0, and Tektin-4) and fertilization-associated gene (SP18 kDa).

## 2 Materials and Methods

### 2.1 Ethics Statement

Experimental protocols were approved by the Animal Care and Use Committee of Chonnam National University (CNU IACUC-YS-2020-5). All experiments were conducted following the Guidelines for the Care and Use of Laboratory Animals of the National Institutes of Health.

### 2.2 Experimental Reagents

Glucose (G), sucrose (S), trehalose (T), l-ascorbic acid (L-As), dimethyl sulfoxide (DMSO), ethylene glycol (EG), propylene glycol (PG), glycerol (GLY), methanol (MeOH), and JC-1 dye were purchased from Sigma-Aldrich (S. Louis, MO, United States). Phosphate buffered saline (PBS: Ca^2+^ and Mg free) was obtained from Life Technologies Ltd. (Paisley, UK). LysoTracker™ green DND-26, and the LIVE/DEAD^®^ sperm viability kit were bought from Invitrogen Molecular Probes (Eugene, OR, United States). A comet assay^®^ (single-cell gel electrophoresis) kit was purchased from Trevigen Inc. (Gaithersburg, MD, United States).

### 2.3 Animal Handling and Management

Abalone hatchery (Tou-Jong soosan) in Yeosu, South Korea, provided 3-year old mature Pacific abalone (*H. discus hannai*) during the spawning season. Abalones were reared in cemented tanks supplied with continuous seawater and an aeration system. Brown algae, *Saccharina latissima,* were supplied to the tank as food for the abalone. Cleaning was accomplished when required. Abalones were carefully chosen by observing the whitish color of swollen gonads.

A total of 185 abalones (male: n = 155; female: n = 30) were used to conduct the experiments. Inducing and sperm collection were accomplished according to the methods previously described by Hossen et al. (2021a, 2021b). Briefly, abalones were induced in sunlight for 1 hour with the shell facing down and another 30 min with the muscle facing down. Abalones were gently stripped to collect sperm. Sperm samples were immediately transferred to Eppendorf tubes using a disposable plastic dropper and kept in a refrigerator (4°C) until use (no more than 15 min).

### 2.4 Quality Evaluation of Fresh Sperm

Sperm quality indicators such as motility, plasma membrane integrity (PMI), acrosome integrity (AI), mitochondrial membrane potential (MMP), and DNA integrity were assessed to ensure the quality of fresh sperm. Experiments were conducted using sperm having more than 90% motility. Sperm motility was observed according to the method described previously (Kim et al., 2020a; Hossen et al., 2021a; 2021b). Briefly, the sperm were diluted 10 times with filtered seawater (FSW) in an Eppendorf tube. Subsequently, 1 µL of the diluted sample was gently mixed with 50 µL of FSW on a glass slide (Superfrost Plus, microscope slide, Fisher Scientific, United States) and observed under a microscope (Nikon Eclipse E200) using a 20x objective lens. Fresh sperm motility is present as percent value (mean ± SD) (*n* = 10). PMI, AI, MMP, and DNA integrity were determined using LIVE/DEAD^®^_,_ LYSO-G/PI^®^, JC-1 dye, and a comet assay kit^®^ (described in Section 2.6 and Section 2.7), respectively.

### 2.5 Sperm Cryopreservation Protocol

The basic sperm cryopreservation protocol was applied according to the method described previously (Kim et al., 2020a; Hossen et al., 2021a). Briefly, the sperm were diluted with filtered seawater (FSW) at a ratio of 1:10 (sperm:SW). Penetrating cryoprotectant (P-CPA) solutions were prepared by mixing each P-CPA (8% DMSO, 8% EG, 6% PG, 2% GLY, or 2% MeOH) with FSW. Saccharides (glucose, sucrose, and trehalose) or vitamin (l-ascorbic acid) at different concentrations (described in Section 2.5) were separately mixed with P-CPA solutions to prepare final extenders. Diluted sperm were mixed with each extender at an equal ratio (v:v) with a final concentration of 3.85 × 10^7^ cells/mL. Sperm samples were equilibrated for 10 min and transferred into 0.5 ml straws using an Eppendorf syringe. Sealing powder was used to seal the straws. The straws were then positioned in a 5 cm rack height of a Styrofoam box (length: 25.0 cm x width: 25.0 cm x height: 21.0 cm) for 10 min and subsequently submerged into liquid nitrogen for at least 2 hours. The straws were then transferred into a 38-L storage tank (model: 38VHC-11M, serial: 80907, Worthington Industries, United States) until further use. The straws were then thawed at 60°C for 5 s in a water bath (JISICO lab & Scientific Instrument, Seoul, South Korea) using seawater.

### 2.6 Effects of Saccharides and Vitamin on Post-Thaw Sperm Motility

To determine the effects of saccharides and vitamin combined with penetrating CPAs, five types of penetrating CPAs at suitable concentrations (8% DMSO, 8% EG, 6% PG, 2% GLY, or 2% MeOH) were selected based on our previous findings (Kim et al., 2020a). Three saccharides, glucose (G), sucrose (S), and trehalose (T), and a vitamin, l-ascorbic acid (L-As), were also selected to conduct the experiment (*n* = 10).

#### 2.6.1 Experiment-1: Effects of Saccharides + DMSO or Vitamin + DMSO on Post-Thaw Sperm Motility

Saccharides were separately mixed with 8% DMSO at different final concentrations (1, 2, 3, 4, and 5%). L-As was mixed with 8% DMSO at final concentrations of 0.1, 0.2, 0.3, 0.4, and 0.5%.

#### 2.6.2 Experiment-2: Effects of Saccharides + EG or Vitamin + EG on Post-Thaw Sperm Motility

Saccharides were separately mixed with 8% EG at different final concentrations (1, 2, 3, 4, and 5%). L-As was supplemented with 8% EG at final concentrations of 0.1, 0.2, 0.3, 0.4, and 0.5%.

#### 2.6.3 Experiment-3: Effects of Saccharides + PG or Vitamin + PG on Post-Thaw Sperm Motility

Saccharides were separately mixed with 6% PG at different final concentrations (1, 2, 3, 4, and 5%). L-As was supplemented with 6% PG at final concentrations of 0.1, 0.2, 0.3, 0.4, and 0.5%.

#### 2.6.4 Experiment-4: Effects of Saccharides + GLY or Vitamin + GLY on Post-Thaw Sperm Motility

Saccharides were separately mixed with 2% GLY at different final concentrations (1, 2, 3, 4, and 5%). L-As was supplemented with each penetrating CPA at final concentrations of 0.1, 0.2, 0.3, 0.4, and 0.5%.

#### 2.6.5 Experiment-5: Effects of Saccharides + MeOH or Vitamin + MeOH on Post-Thaw Sperm Motility

Saccharides were separately mixed with 2% MeOH at different final concentrations (1, 2, 3, 4, and 5%). L-As was supplemented with 2% MeOH at final concentrations of 0.1, 0.2, 0.3, 0.4, and 0.5%.

### 2.7 Fluorescence Technique to Assess Post-Thaw Sperm Quality

Five cryoprotectant solutions (3% S + 8% DMSO, 1% G + 8% EG, 2% G + 6% PG, 3% G + 2% GLY, and 4% T + 2% MeOH) were selected to assess the post-thaw sperm quality based on the best findings from each motility experiment.

#### 2.7.1 Plasma Membrane Integrity (PMI)

Plasma membrane integrity (PMI) was visualized using a LIVE/DEAD^®^ sperm viability kit following the protocol described by Hossen et al. (2021a) with slight modifications. Briefly, the sperm samples were diluted with FSW to a final concentration of 1 × 10^6^ cells/mL. Diluted sperm samples were fixed using a hemocytometer (Paul Marienfeld GmbH & Co., Germany). A 2.5 µL aliquot of SYBR™ 14 dye was mixed with 500 μL of the sperm sample and incubated at 37°C for 10 min in the dark. Subsequently, PI (5 µL) was gently mixed with each sample for further incubation at 37°C for 10 min in the dark. A 2 μL aliquot of the stained sample was placed on a glass slide and visualized under a fluorescence microscope (Nikon Eclipse E600, Japan). SYBR14-stained images of intact plasma membranes (SYBR14^+^/PI^−^) were captured using a green filter (excitation wavelength: 450–490 nm). PI-stained images of damaged plasma membranes (SYBR14^-^/PI^+^) were captured using a red filter (emission wavelength: 510–560 nm). Visualization and analysis (*n* = 10) were performed according to the method described by Hossen et al. (2021c).

#### 2.7.2 Acrosome Integrity (AI)

Acrosome integrity of fresh and cryopreserved sperm was determined using a previously described LYSO-G/PI^®^ method (Hossen et al., 2021a; Hossen et al., 2021b) with slight modifications. Briefly, the sperm were diluted with FSW to a final concentration of 1 × 10^6^ cells/mL. LYSO-G and PI dye were pre-incubated in a thermobath (model: ALB128, FINEPCR^®^, Korea) at 37°C for 10 min. After that, 5 µL of LYSO-G and 10 µL of PI were gently mixed with 500 µL of the sample and incubated at 37°C for 30 min in the dark. The stained sample (2 µL) was placed on a glass slide and covered with a cover slip. Samples were instantly observed under a fluorescence microscope (Nikon Eclipse E600, Japan). LYSO-G-stained images of intact acrosomes (LYSO-G^+^/PI^−^) were captured using a green filter (B-2A; Ex 450–490 nm). PI-stained images of damaged spermatozoa were captured using a red filter (G-2A; Ex 510–560 nm). Fluorescence images captured with green and red filters were merged with pictures taken without a filter to determine the AI values of fresh and cryopreserved sperm. Visualization and analysis (*n* = 10) were performed according to the method described in the “plasma membrane integrity” section.

#### 2.7.3 Mitochondrial Membrane Potential (MMP)

Mitochondrial membrane potential (MMP) values of fresh and cryopreserved sperm were detected using a previously described JC-1 fluorescent dye method (Binet et al., 2014), with minor modifications. Briefly, 2.5 μL of JC-1 dye was mixed with 300 μL of the sample (1 × 10^6^ cells/mL) and incubated at 37°C in the dark for 18 min. Subsequently, JC-1-stained images of intact mitochondrial membranes (red) were captured using a red filter (G-2A; ex: 510–560 nm). The fluorescence images captured with the red filter were merged with the pictures taken without a filter to detect the MMP values of fresh and cryopreserved sperm. Sperm having a red colored mitochondrial membrane was considered intact mitochondria. Visualization and analysis (*n* = 10) were performed according to the method described in the previous section.

### 2.8 Comet Assay to Detect DNA Integrity of Sperm

Comet assays (single-cell gel electrophoresis) of sperm cryopreserved using 3% S + 8% DMSO, 1% G + 8% EG, 2% G + 6% PG, 3% G + 2% GLY, and 4% T + 2% MeOH were performed using a Comet assay^®^ kit following a published protocol (Hossen et al., 2021a) with minor modifications. Briefly, fresh and post-thaw sperm were diluted with pre-chilled 1× PBS to a final concentration of 1 × 10^5^ cells/mL. the sperm were immobilized on comet slides™ using comet agarose. The slides were treated with pre-chilled lysis buffer solution for 1 hour and then treated with pre-chilled alkaline unwinding solution for another hour. After that, the slides were electrophoresed in a cometAssay^®^ electrophoresis system for 30 min at 21 V with an alkaline electrophoresis solution. The slides were washed twice with distilled deionized water, dried in a 37°C incubator for 30 min, and stained with Vista Green DNA dye. Stained comets were visualized and captured using a fluorescence microscope (Ex 450–490 nm, Nikon Eclipse E600, Japan) with a 20× lens. A minimum of 100 comets from each replication was used to analyze the comet parameters (n = 5). Sperm DNA integrity parameters such as % DNA in tail and olive tail moment were analyzed using comet Assay IV image analysis software (version 4.3.2, Perceptive Instruments Ltd., UK).

### 2.9 Adenosine Triphosphate (ATP) Assay

Intracellular adenosine triphosphate (ATP) contents in fresh and cryopreserved sperm were detected using an ATP assay kit (code # BM-ATP-100, PicoSens™, Biomax, Seoul, South Korea). Briefly, sperm samples were homogenized in 100 μL of assay buffer and later resuspended in assay buffer to a concentration of 3.87 × 10^6^ cells/mL. Then, 50 μL of each sample (*n* = 10) was transferred to a well in a 96-well plate. Subsequently, 50 μL of the ATP reaction mixture was added to each well and mixed by gently shaking the plate for 2 min in a rotary shaker to induce cell lysis. After 30 min of incubation at room temperature in the dark, the absorbance at 570 nm was measured using a microplate reader (Epoch 2, BioTek, Winooski, VT, United States). To generate a standard curve, ATP standard solutions were prepared from a 10 mM ATP standard. The absorbance value for each test sample was converted to the corresponding ATP concentration (nM) using the standard curve.

### 2.10 mRNA Content of Motility-Associated Genes in Sperm Cryopreserved Using Saccharides Supplemented With Penetrating CPAs

After the sperm were cryopreserved with different cryoprotectant solutions (3% S + 8% DMSO, 1% G + 8% EG, 2% G + 6% PG, 3% G + 2% GLY, and 4% T + 2% MeOH), the mRNA content of motility and fertilization-associated genes was quantified (*n* = 5).

#### 2.10.1 Total RNA Extraction and cDNA Synthesis of Cryopreserved Sperm

Total RNAs were extracted from fresh and cryopreserved (3% S + 8% DMSO, 1% G + 8% EG, 2% G + 6% PG, 3% G + 2% GLY, and 4% T + 2% MeOH) sperm (*n* = 5). An RNeasy mini kit (Qiagen, Hilden, Germany) was used to extract the total RNA according to the method described by Hossen et al. (2021a; 2021b). Genomic DNA contamination was eliminated by performing RNase-free DNase (Promega, Madison, WI, United States) treatment. Concentrations of the total RNA were measured with a spectrophotometer (ACTGene ASP-2680, United States). The Total RNA was reverse transcribed to cDNAs using a Superscript^®^ III First-Strand synthesis kit (Invitrogen, Carlsbad, CA, United States).

#### 2.10.2 Quantitative Real-Time PCR

Quantitative PCR (qRT-PCR) was performed to determine the mRNA expression levels of HSP70, HSP90, PKA-C, Axp66.0, Tektin-4, and SP18 kDa in fresh and cryopreserved (3% S + 8% DMSO, 1% G + 8% EG, 2% G + 6% PG, 3% G + 2% GLY, and 4% T + 2% MeOH) sperm samples. Gene-specific primers (Table 1) were designed to perform qRT-PCR in a LightCycler^®^ 96 System (Roche, Germany) using a 2× qPCRBIO SyGreen Mix Lo-Rox kit (PCR Biosystems, Ltd., London, UK) according to published methods (Hossen et al., 2021a; Hossen et al., 2021b). Briefly, a 20 µL reaction mix containing 1 µL cDNA template of each sperm sample, 1 µL (10 pmol) of each forward and reverse primer, 10 µL SyGreen Mix, and 7 µL PCR-grade water were used to perform qRT-PCR. The melting temperature was determined using a default setting: 95°C for 10 s, 65°C for 1 min, and 97°C for 1 min. PCR conditions were fixed according to conditions described by Hossen et al. (2021a). The relative mRNA content was quantified using the 2^−ΔΔct^ method (Livak and Schmittgen, 2001). mRNA contents were normalized against the expression level of β-actin, a housekeeping gene.

**TABLE 1:** List of primers used for qRT-PCR quantification of genes in sperm.

Gene	Primer	Sequence	Amplicon length (Bp)	GenBank/References
β-actin	Sense	5′-CCG​TGA​AAA​GAT​GAC​CCA​GA-3′	204	AY380809.1
	Antisense	5′-TAC​GAC​CGG​AAG​CGT​ACA​GA-3′		
HSP70	Sense	5′-CAG​AGA​ACA​CAA​TCT​TCG​ATG​C-3′	277	DQ324856.1
	Antisense	5′-CGT​TGA​GAG​TCG​TTG​AAG​TAA​G-3′		
HSP90	Sense	5′-AAC​AGT​ACA​TCT​GGG​AGT​CG-3′	216	GU014545.1
	Antisense	5′-CCT​CCT​TGT​CTC​TTT​CCT​TCT-3′		
PKA-C	Sense	5′-AGC​CAG​CAG​TTG​CAA​ATG​C-3′	199	Kong et al. (2020)
	Antisense	5′-CTT​CTC​ATT​TAA​TGT​GTG​CTC​C-3′		
Axp66.0	Sense	5′-GGT​CAA​GTT​CAA​CAA​CCA​GC-3′	200	MN270935.1
	Antisense	5′-GCA​TCT​TGT​TGT​ACG​CCT​CG-3′		
Tektin-4	Sense	5′-TCC​GAG​GTG​ACC​AAG​AAG​C-3′	185	MZ265399.1
	Antisense	5′-CAG​TTC​AGA​TTG​TCT​GTT​GCA-3′		
SP18 kDa	Sense	5′-GTA​TCC​GCA​ATG​AAG​GTA​GGG-3′	194	OL411494.1
	Antisense	5′-CCT​CTC​GCC​TTT​ATC​ATC​AG-3′		

### 2.11 Fertility and Hatchability Test

Reproductively matured females (N = 30) were selected from the rearing tank. Induction of spawning was accomplished according to a method described by Hossen et al. (2021a). After spawning, the egg quality was checked under a microscope (Nikon Eclipse E200, Japan) to evaluate the shape of the envelope, the nucleus, and egg color. Sperm cryopreserved with 8% DMSO or 8% DMSO combined with 3% sucrose were used in *in vitro* fertilization to observe the fertilization and hatching rates. Fertilization experiments were conducted using a series of plastic bowls (2 L, 40,000 eggs in each bowl). The sperm to egg ratio of 10,000:1 was maintained in the fertilization experiment according to a previous report (Hossen et al., 2021a). Fertilized eggs were washed three times (30 min intervals) using FSW. The water temperatures of experimental bowls were maintained at 18–20 °C. The fertilization rate (*n* = 3) was calculated based on a 2 h post-fertilized embryo. The hatching rate (*n* = 3) was analyzed based on 16 h post-fertilized veliger larvae. The fertilization rate and hatching rate were calculated based on the method described by Hossen et al. (2021a).

### 2.12 Statistical Analysis

All statistical analyses were performed using SPSS 16.00 (SPSS Inc., Chicago, IL, United States). All statistical data generated in figures and tables are presented as mean ± standard deviation (SD). One-way analysis of variance (ANOVA) and Duncan’s multiple comparisons test were used to evaluate different treatments. Differences were considered statistically significant at *p* < 0.05. Pearson correlation analysis was performed to determine the relationships between sperm quality with oxidative stress-associated parameters. GraphPad Prism software (GraphPad Prism version 9.3.1 for Windows; GraphPad Software, CA, United States) was used to generate graphs. Pearson’s correlation coefficient was determined using SPSS 16.00 with the standard procedure. Pearson’s correlation coefficient was determined using a standard procedure in SPSS 26.00. The correlation was considered significant at the 0.01 level (two-tailed).

## 3 Results

### 3.1 Experiment-1: Effects of Saccharides + DMSO or Vitamin + DMSO on Post-Thaw Sperm Motility

**FIGURE 1:**
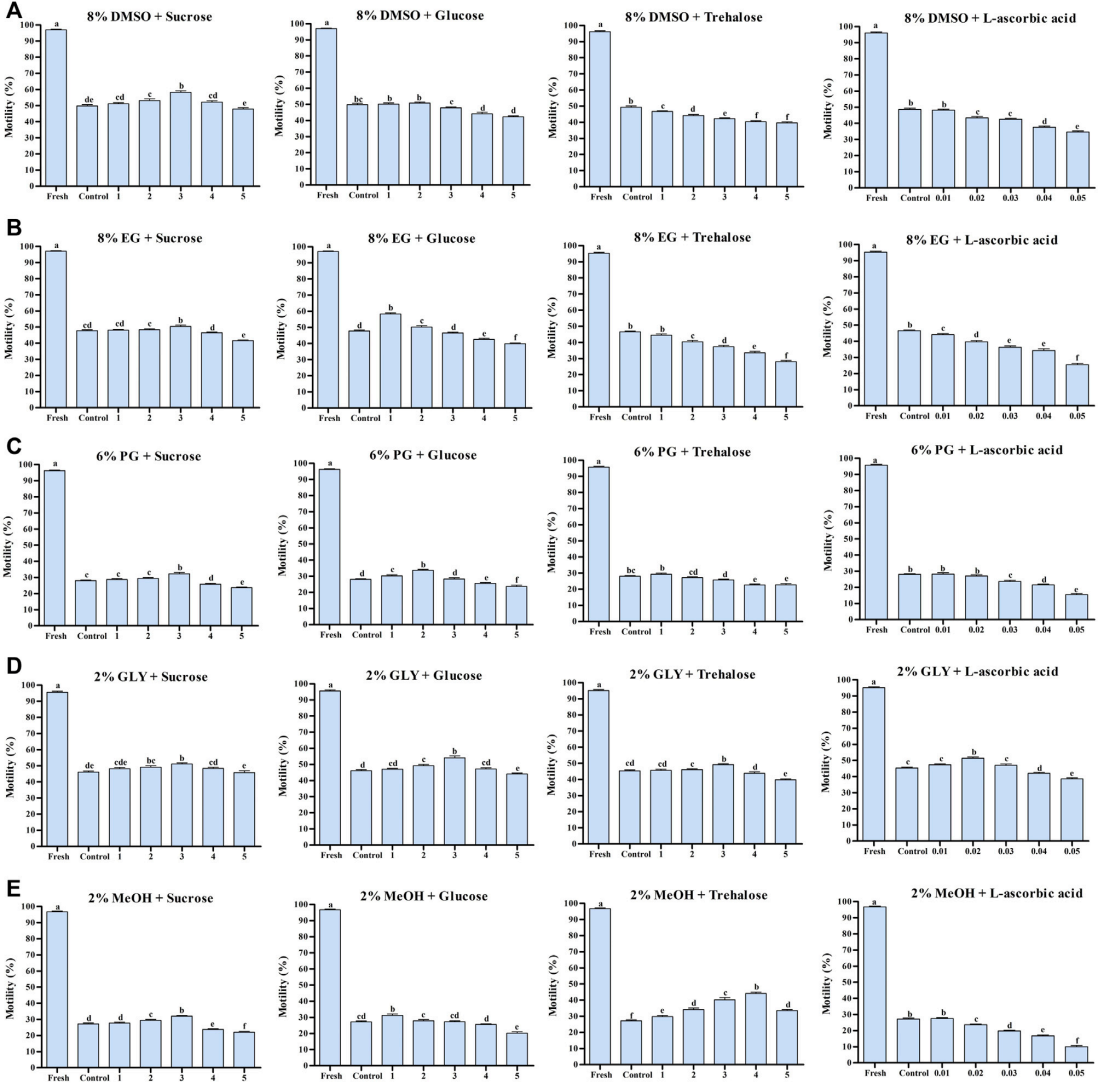
Effects of saccharides (sucrose, glucose, and trehalose) and vitamin (l-ascorbic acid) on the post-thaw sperm quality of Pacific abalone. **(A)** Post-thaw motility of sperm cryopreserved using different concentrations of saccharides (1, 2, 3, 4, and 5%) and vitamin (0.01, 0.02, 0.03, 0.04, and 0.05%) combined with 8% DMSO. **(B)** Post-thaw motility of sperm cryopreserved using different concentrations of saccharides (1, 2, 3, 4, and 5%) and vitamin (0.01, 0.02, 0.03, 0.04, and 0.05%) combined with 8% EG. **(C)** Post-thaw motility of sperm cryopreserved using different concentrations of saccharides (1, 2, 3, 4, and 5%) and vitamin (0.01, 0.02, 0.03, 0.04, and 0.05%) combined with 6% PG. **(D)** Post-thaw motility of sperm cryopreserved using different concentrations of saccharides (1, 2, 3, 4, and 5%) and vitamin (0.01, 0.02, 0.03, 0.04, and 0.05%) combined with 2% GLY. **(E)** Post-thaw motility of sperm cryopreserved using different concentrations of saccharides (1, 2, 3, 4, and 5%) and vitamin (0.01, 0.02, 0.03, 0.04, and 0.05%) combined with 2% MeOH. Significant difference (*p* < 0.05) is denoted by different letters.

Sperm cryopreserved using 3% S + 8% DMSO showed significantly higher post-thaw motility (58.4 ± 2.9%) than sperm in other groups (Figure 1A). Supplementation of L-As with 8% DMSO did not improve the post-thaw motility (Figure 1A).

### 3.2 Experiment-2: Effects of Saccharides + EG or Vitamin + EG on Post-Thaw Sperm Motility

The highest post-thaw motility was detected when the sperm were cryopreserved using 1% G + 8% EG (58.3 ± 2.1%) (Figure 1B). However, cryopreservation with 3% S + 8% EG improved the post-thaw motility compared with the control. Notably, L-As with 8% EG did not improve post-thaw motility (Figure 1B).

### 3.3 Experiment-3: Effects of Saccharides + PG or Vitamin + PG on Post-Thaw Sperm Motility

Sperm cryopreserved with 2% G + 6% PG had the highest post-thaw sperm motility (33.7 ± 1.8%) (Figure 1C). Sperm cryopreserved with 2% G + 6% PG also showed significantly improved post-thaw motility compared with the control. However, L-As with 8% EG did not improve post-thaw motility compared with the control (Figure 1C).

### 3.4 Experiment-4: Effects of Saccharides + GLY or Vitamin + GLY on Post-Thaw Sperm Motility

In this experiment 3% G, 3% T, and 0.2% L-As individually combined with 2% GLY significantly improved the post-thaw sperm motility (Figure 1D). Sperm cryopreserved with 3% G + 2% GLY showed the highest post-thaw motility (54.1 ± 3.8%).

### 3.5 Experiment-5: Effects of Saccharides + MeOH and Vitamin + MeOH on Post-Thaw Sperm Motility

In this experiment, 3% S, 1% G, or 4% T combined with 2% MeOH significantly improved the post-thaw sperm motility (Figure 1E). Sperm cryopreserved with 4% T + 2% MeOH showed the highest post-thaw motility (44.1 ± 2.8%).

### 3.6 Fluorescent Technique for Assessing PMI, AI, and MMP of Cryopreserved Sperm

#### 3.6.1 Plasma Membrane Integrity (PMI)

**FIGURE 2:**
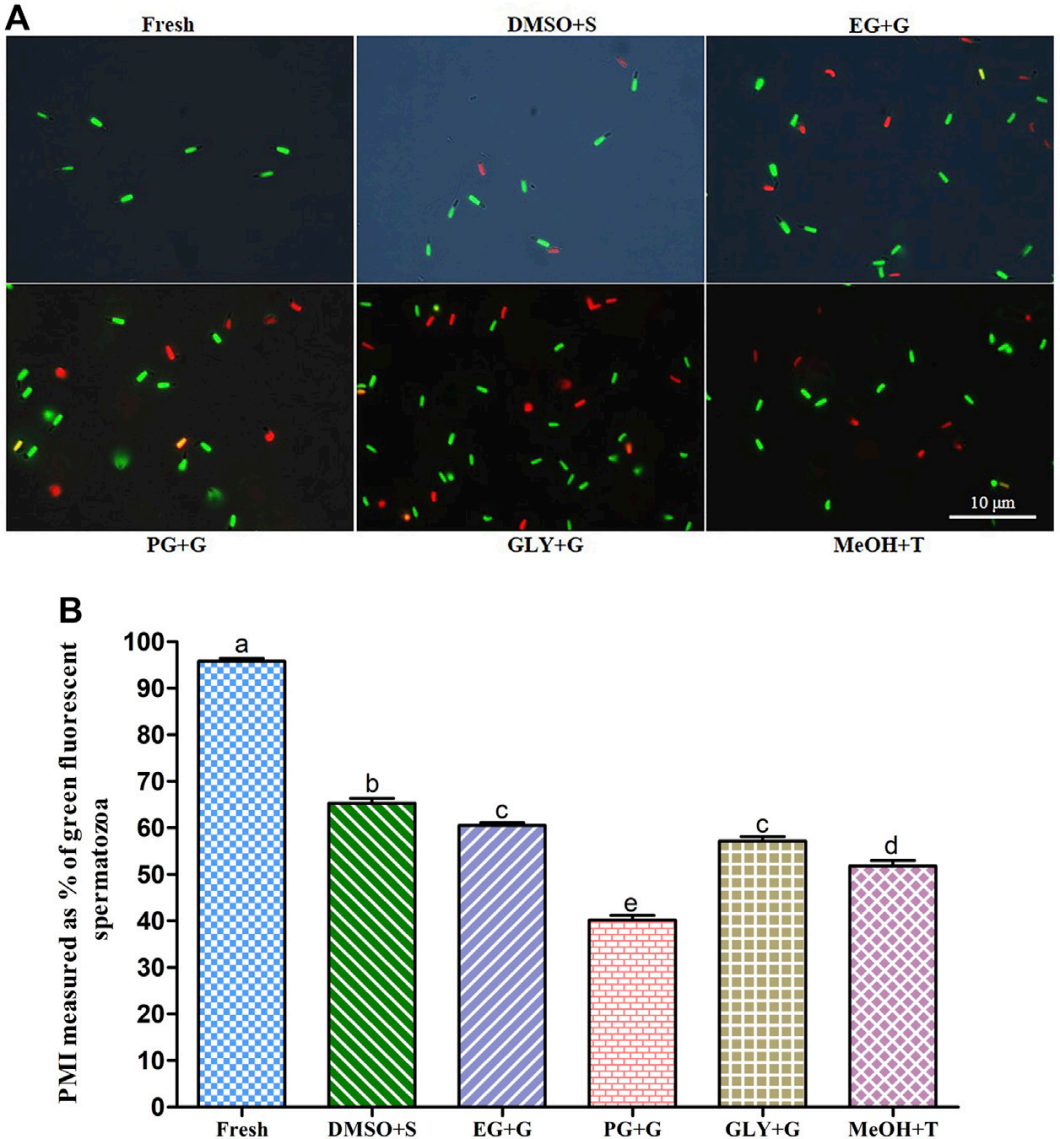
Effects of saccharides (sucrose, glucose, and trehalose) on plasma membrane integrity (PMI) of post-thaw sperm. **(A)** SYBR14/PI-stained images of fresh and cryopreserved sperm. **(B)** PMI values of different types of post-thaw sperm. Results are presented as mean values ±standard deviation (*n* = 10). DMSO + S: 8% dimethyl sulfoxide (DMSO) combined with 3% sucrose (S), EG + G: 8% ethylene glycol (EG) combined with 1% glucose (G), PG + G: 6% propylene glycol (PG) combined with 2% glucose (G), GLY + G: 2% glycerol combined with 3% glucose (G), and MeOH + T: 2% methanol (MeOH) combined with 4% trehalose (T). Significantly different levels (*p* < 0.05) are denoted by different letters.

Sperm cryopreserved using 3% S + 8% DMSO showed significantly (*p* < 0.05) higher plasma membrane integrity (PMI) (65.3 ± 3.3%) than sperm cryopreserved with other types of cryoprotectant solutions (Figure 2). However, the PMI of sperm cryopreserved using 1% G + 8% EG (60.5 ± 1.8%) was not significantly different from that of the sperm cryopreserved using 3% G + 2% GLY (57.2 ± 2.9%) (*p* > 0.05).

#### 3.6.2 Acrosome Integrity (AI)

**FIGURE 3:**
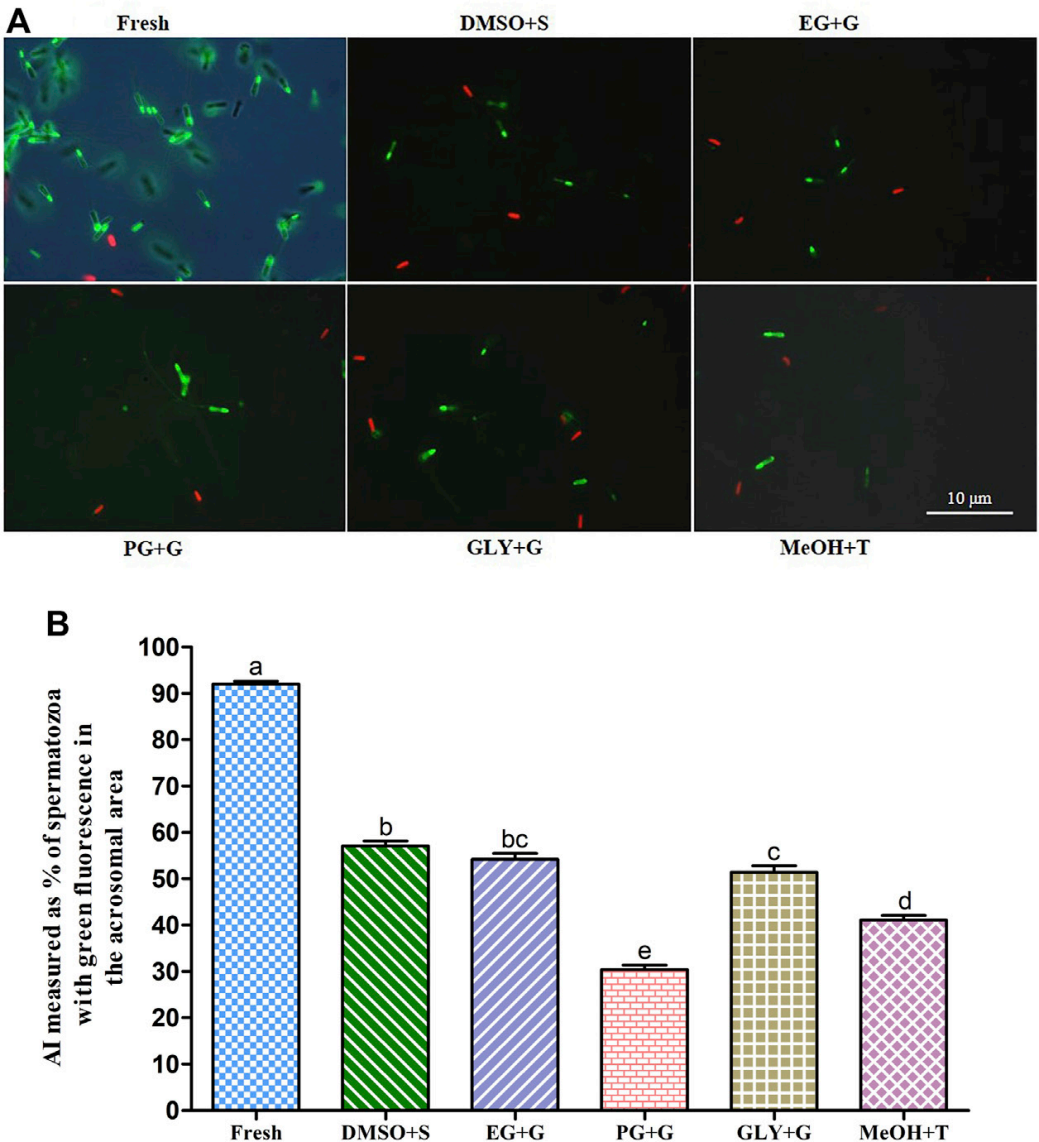
Effects of saccharides (sucrose, glucose, and trehalose) on acrosome integrity (AI) of post-thaw sperm. **(A)** LYSO-G/PI-stained images of fresh and cryopreserved sperm. **(B)** AI values of different types of post-thaw sperm. Results are presented as mean values ±standard deviation (*n* = 10). DMSO + S: 8% dimethyl sulfoxide (DMSO) combined with 3% sucrose (S), EG + G: 8% ethylene glycol (EG) combined with 1% glucose (G), PG + G: 6% propylene glycol (PG) combined with 2% glucose (G), GLY + G: 2% glycerol combined with 3% glucose (G), and MeOH + T: 2% methanol (MeOH) combined with 4% trehalose (T). Significantly different levels (*p* < 0.05) are denoted by different letters.

Sperm cryopreserved using 3% S + 8% DMSO showed improved acrosome integrity (AI) (57.1 ± 3.2%) than sperm cryopreserved with other types of cryoprotectant solutions (Figure 3). However, the sperm cryopreserved using 2% G + 6% PG showed significantly (*p* < 0.05) lower AI (30.4 ± 2.9%) than sperm cryopreserved with other types of cryoprotectant solutions.

#### 3.6.3 Mitochondrial Membrane Potential (MMP)

**FIGURE 4:**
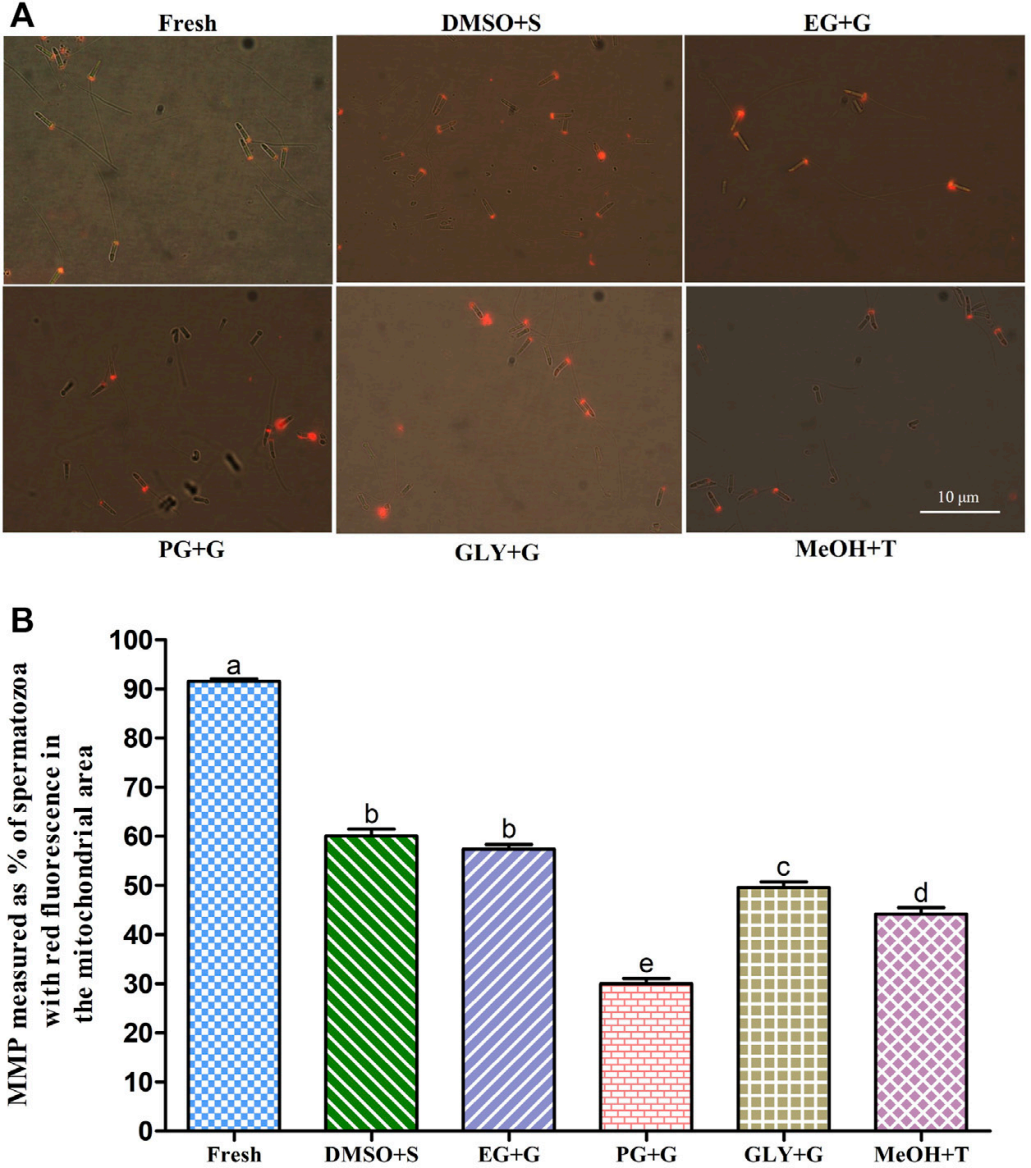
Effects of saccharides (sucrose, glucose, and trehalose) on the mitochondrial membrane potential (MMP) of post-thaw sperm. **(A)** JC-1-stained images of fresh and cryopreserved sperm. **(B)** MMP results of different types of post-thaw sperm. Results are presented as mean values ±standard deviation (n = 10). DMSO + S: 8% dimethyl sulfoxide (DMSO) combined with 3% sucrose (S), EG + G: 8% ethylene glycol (EG) combined with 1% glucose (G), PG + G: 6% propylene glycol (PG) combined with 2% glucose (G), GLY + G: 2% glycerol combined with 3% glucose (G), and MeOH + T: 2% methanol (MeOH) combined with 4% trehalose (T). Significantly different levels (*p* < 0.05) are denoted by different letters.

Sperm cryopreserved using 3% S + 8% DMSO showed an improved mitochondrial membrane potential (MMP) **(**60.1 ± 4.3%) than sperm cryopreserved with other types of cryoprotectant solutions (Figure 4). However, sperm cryopreserved using 2% G + 6% PG showed a significantly (*p* < 0.05) lower MMP (30.0 ± 3.4%) than sperm cryopreserved with other types of cryoprotectant solutions.

### 3.7 Sperm DNA Integrity

**FIGURE 5:**
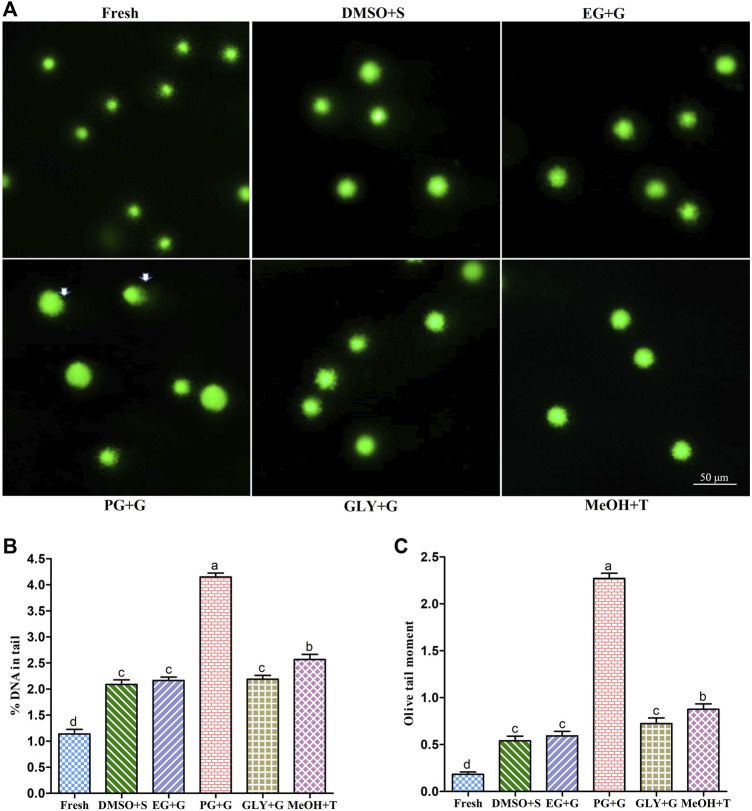
Deoxyribonucleic acid (DNA) integrity of sperm cryopreserved using saccharides (sucrose, glucose, and trehalose). **(A)** Vista green dye-stained comet images of fresh and cryopreserved sperm. **(B)** Sperm nuclear DNA fragmentation (% DNA in tail) in different types of post-thaw sperm. **(C)** Olive tail moment of different types of post-thaw sperm. Results are presented as mean values ±standard deviation (n = 5). DMSO + S: 8% dimethyl sulfoxide (DMSO) combined with 3% sucrose (S), EG + G: 8% ethylene glycol (EG) combined with 1% glucose (G), PG + G: 6% propylene glycol (PG) combined with 2% glucose (G), GLY + G: 2% glycerol combined with 3% glucose (G), and MeOH + T: 2% methanol (MeOH) combined with 4% trehalose (T). Significantly different levels (*p* < 0.05) are denoted by different letters.

Results of deoxyribonucleic acid (DNA) integrity of different types of post-thaw sperm are shown in Figure 5. The % DNA in tail of sperm cryopreserved with 3% S + 8% DMSO was 2.09 ± 0.20%, which was not significantly (*p* > 0.05) different from that of sperm cryopreserved using 1% G + 8% EG (2.16 ± 0.15%) or 3% G + 2% GLY (2.19 ± 0.17%).

### 3.8 Adenosine Triphosphate (ATP) Contents

**FIGURE 6:**
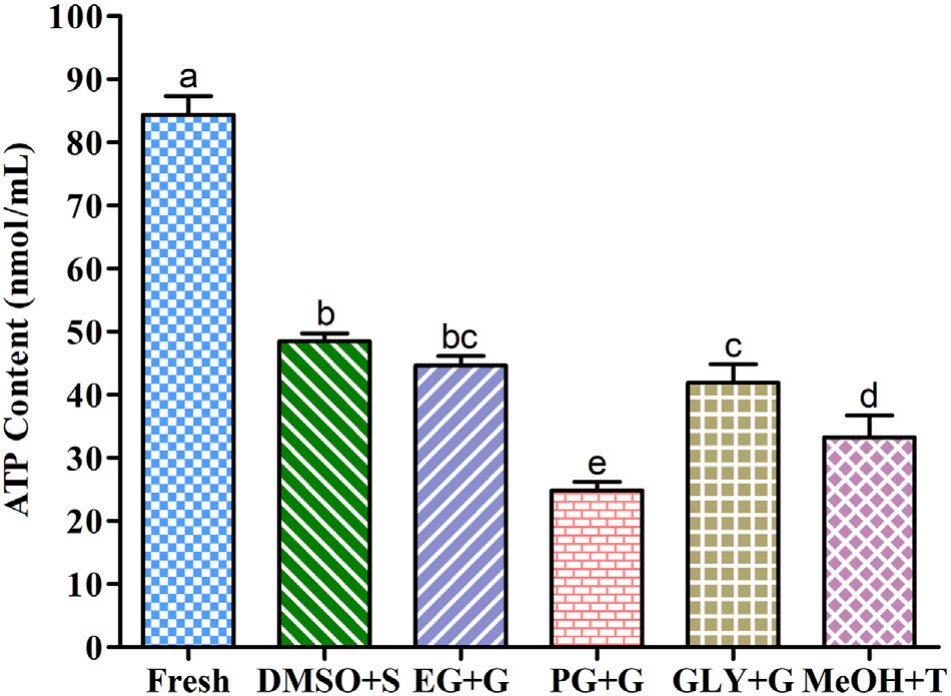
Adenosine triphosphate (ATP) contents in different types of post-thaw sperm cryopreserved using saccharides. DMSO + S: 8% dimethyl sulfoxide (DMSO) combined with 3% sucrose (S), EG + G: 8% ethylene glycol (EG) combined with 1% glucose (G), PG + G: 6% propylene glycol (PG) combined with 2% glucose (G), GLY + G; 2% glycerol combined with 3% glucose (G), and MeOH + T: 2% methanol (MeOH) combined with 4% trehalose (T). Results are presented as mean values ±standard deviation (*n* = 10). Significantly different levels (*p* < 0.05) are denoted by different letters.

Sperm cryopreserved using saccharides (Figure 6) showed improved intracellular ATP concentrations than those cryopreserved without the addition of saccharides (Supplementary Figure S1). Sperm cryopreserved using 3% sucrose combined with 8% DMSO presented the highest intracellular ATP content among all cryopreserved sperm. Sperm cryopreserved using 2% glucose combined with 6% PG showed a significantly lower ATP content than other examined cryopreserved sperm samples. However, fresh sperm showed significantly higher ATP contents than all examined cryopreserved sperm.

### 3.9 Gene Expression in Cryopreserved Sperm

#### 3.9.1 Expression of Motility Regulating Gene in Cryopreserved Sperm

##### 3.9.1.1 Expression Analysis of the HSP70 mRNA Transcript

**FIGURE 7:**
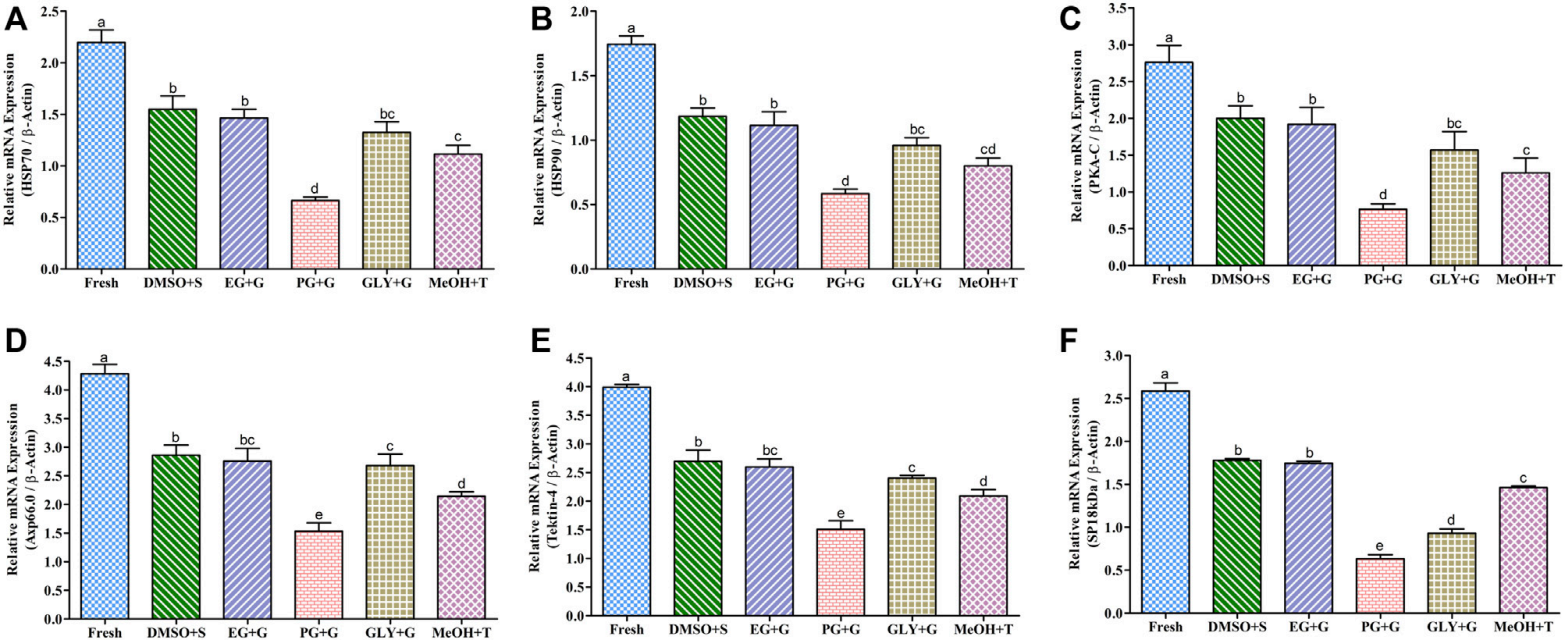
mRNA content of motility and fertility-associated gene in saccharide-supplemented cryopreserved sperm of Pacific abalone (n = 5). **(A)** Heat shock protein 70 (HSP70) mRNA content. **(B)** Heat shock protein 90 (HSP90) mRNA content. **(C)** cAMP-dependent protein kinase (PKA-C) mRNA content. **(D)** Axonemal protein 66.0 (Axp66.0) mRNA content. **(E)** Tektin-4 mRNA content. **(F)** Fertilization protein (SP18 kDa) mRNA content. mRNA content values were normalized against average ΔCT values of the control. DMSO + S: 8% dimethyl sulfoxide (DMSO) combined with 3% sucrose (S), EG + G: 8% ethylene glycol (EG) combined with 1% glucose (G), PG + G: 6% propylene glycol (PG) combined with 2% glucose (G), GLY + G: 2% glycerol combined with 3% glucose (G), and MeOH + T: 2% methanol (MeOH) combined with 4% trehalose (T). Significantly different levels (*p* < 0.05) are denoted by different letters.

The relative mRNA content of HSP70 in fresh and cryopreserved sperm is given in Figure 7A. Sperm cryopreserved using 3% sucrose combined with 8% DMSO showed improved mRNA content than other types of post-thaw sperm, although the mRNA content of sperm cryopreserved using 3% sucrose combined with 8% DMSO was not significantly different (*p* > 0.05) from that of sperm cryopreserved using 1% glucose combined with 8% EG. However, sperm cryopreserved using 2% glucose combined with 6% PG showed significantly lower mRNA content of HSP70 than other cryopreserved sperm.

##### 3.9.1.2 Expression Analysis of the HSP90 mRNA Transcript

Post-thaw sperm showed significantly (*p* < 0.05) lower HSP90 mRNA content than fresh sperm. The mRNA content of HSP90 in sperm cryopreserved using 3% sucrose combined with 8% DMSO was improved than those in sperm cryopreserved using other types of CPAs (Figure 7B). However, the HSP90 mRNA content of sperm cryopreserved using 3% sucrose combined with 8% DMSO was not significantly (*p* > 0.05) different from that of sperm cryopreserved using 1% glucose combined with 8% EG.

##### 3.9.1.3 Expression Analysis of the PKA-C mRNA Transcript

Sperm cryopreserved using 3% sucrose combined with 8% DMSO showed a higher PKA-C mRNA content than those cryopreserved using other types of cryoprotectants (Figure 7C), except for those cryopreserved using 1% glucose combined with 8% EG which showed no significant (*p* > 0.05) difference in the PKA-C mRNA content with sperm cryopreserved using 3% sucrose combined with 8% DMSO. However, significantly (*p* < 0.05) lower mRNA content of PKA-C was found for post-thaw sperm cryopreserved using 2% glucose combined with 6% PG.

##### 3.9.1.4 Expression Analysis of the Axp66.0 mRNA Transcript

Cryopreserved sperm showed relatively lower Axp66.0 mRNA content than fresh sperm (Figure 7D). However, the Axp66.0 mRNA content in sperm cryopreserved using 3% sucrose combined with 8% DMSO showed significant improvement (*p* < 0.05) than those cryopreserved using other types of cryoprotectants. Post-thaw sperm cryopreserved using 2% glucose combined with 6% PG showed significantly (*p* < 0.05) lower mRNA content of Axp66.0.

##### 3.9.1.5 Expression Analysis of the Tektin-4 mRNA Transcript

Tektin-4 mRNA in cryopreserved sperm was relatively lower than that in fresh sperm (Figure 7E). Sperm cryopreserved using 3% sucrose combined with 8% DMSO showed improved Tektin-4 mRNA content than those cryopreserved using other types of cryoprotectant. However, significantly (*p* < 0.05) lower mRNA expression of tektin-4 was quantified from post-thaw sperm cryopreserved using 2% glucose combined with 6% PG.

#### 3.9.2 Expression of a Fertilization Protein (SP18-kDa) in Cryopreserved Sperm

The SP18-kDa mRNA content was comparatively lower in cryopreserved sperm than in fresh sperm (Figure 7F). However, sperm cryopreserved using 3% S + 8% DMSO or 1% G + 8% EG showed significantly higher expression than other examined cryopreserved sperm (Figure 7F).

### 3.10 Correlations Among Sperm Quality Parameters

Correlations among post-thaw sperm quality parameters are presented in Table 2. Post-thaw sperm motility showed strongly positive correlations with AI (r = 0.917; *p* < 0.01) and MMP (r = 0.913; *p* < 0.01). However, post-thaw motility showed moderately negative correlations with % DNA in tail (r = -0.879; *p* < 0.01).

**TABLE 2:** Correlation among post-thaw sperm quality parameters of Pacific abalone.

	Motility	PMI	AI	MMP	% DNA in tail
PMI	0.888^**^	1	—	—	—
AI	0.917^**^	0.885^**^	1	—	—
MMP	0.882^**^	0.914^**^	0.904^**^	1	—
DNA fragmentation	−0.879^**^	−0.868^**^	−0.857^**^	−0.865^**^	1
ATP	0.898^**^	0.864^**^	0.882^**^	0.879^**^	−0.841^**^

** Correlation is significant at the 0.01 level (2-tailed).

### 3.11 Fertilization and Hatching Rates of Cryopreserved Sperm

**FIGURE 8:**
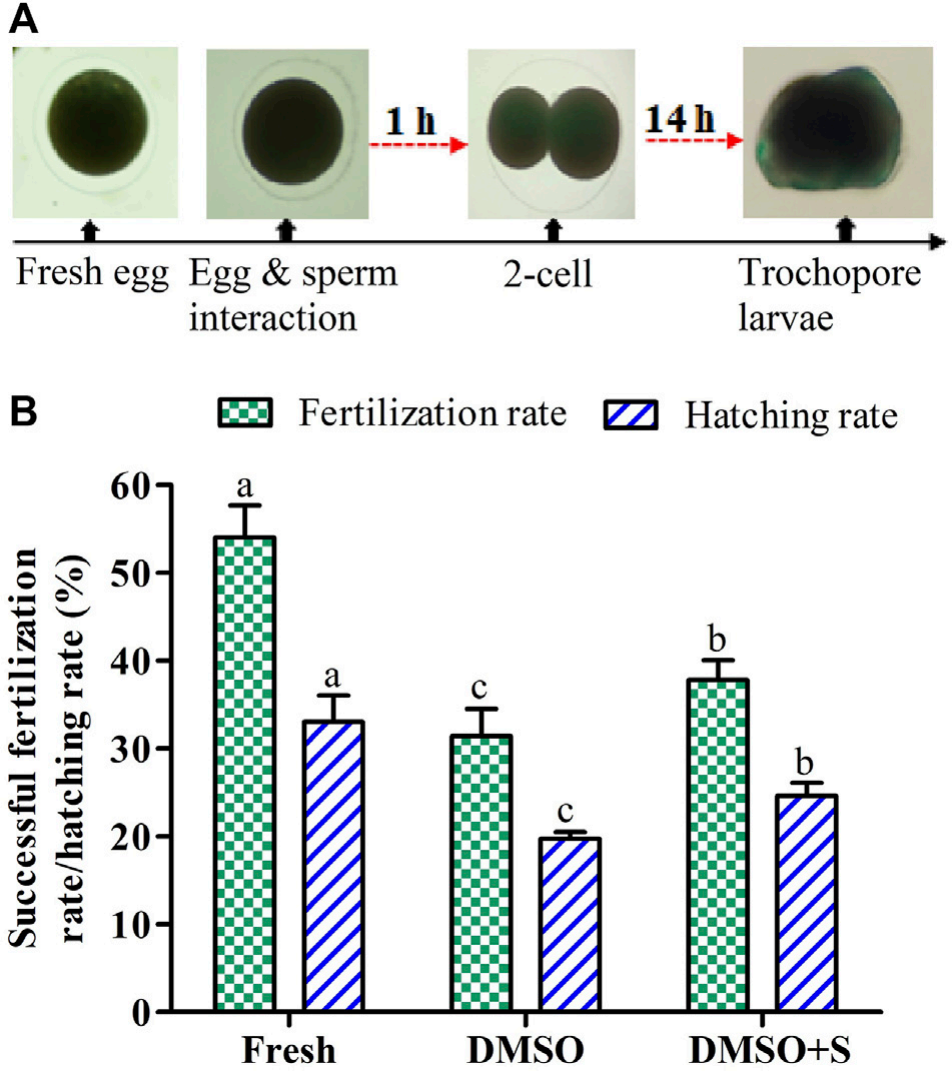
Effects of sucrose on the fertilization and hatching capacity of post-thaw sperm of Pacific abalone (*n* = 3). **(A)** Two-cell division occurred at 1 hour post-fertilization and trochophore larvae hatched out at 14 h post-fertilization. **(B)** Fertility and hatchability of Pacific abalone sperm cryopreserved using 3% sucrose combined with 8% DMSO. DMSO: 8% dimethyl sulfoxide, DMSO + S: 8% dimethyl sulfoxide (DMSO) combined with 3% sucrose (S). Significantly different levels (*p* < 0.05) are denoted by different letters.

Saccharide (sucrose) supplementation with penetrating CPAs improved fertilization and hatching rates than cryopreservation using penetrating CPAs only (Figure 8). However, sperm cryopreserved using 3% sucrose combined with 8% DMSO showed significantly (*p* < 0.05) lower fertilization and hatching rates than fresh sperm.

## 4 Discussion

The goal of the present study was to investigate the effects of saccharides (sucrose, glucose, and trehalose) or vitamin (l-ascorbic acid) with P-CPAs on post-thaw sperm quality (motility, PMI, AI, MMP, DNA integrity, and ATP content), changes in the mRNA content of motility and fertilization-associated genes, fertilization capacity, and hatching capacity of Pacific abalone, *H. discus hannai*. The addition of saccharides or a vitamin to a penetrating CPA is a potential strategy to improve post-thaw sperm quality. It has been used in several aquatic species and marine invertebrates for sperm cryopreservation (Cabrita et al., 2011; Liu et al., 2014a; Liu et al., 2014b; Liu et al., 2016; Rusco et al., 2019; Anjos et al., 2021; Hossen et al., 2021c). The present study revealed that the combination of saccharides with P-CPAs improved post-thaw sperm motility than cryopreservation with only P-CPAs (8% DMSO, 8% EG, 6% PG, 2% GLY, or 2% MeOH). This is likely because saccharides such as glucose and sucrose can stabilize cell membrane phospholipids during the cooling step of cryopreservation (Ahn et al., 2018). On the other hand, trehalose is a non-reducing disaccharide of glucose. It can act as an extracellular CPA by exhibiting a protective role against osmotic effects (Zhu et al., 2017; Ariyan et al., 2021). It can protect the sperm against damage, thereby preventing fertility reduction by protecting ice crystal formation inside the sperm (Purdy, 2006; Ariyan et al., 2021). On the other hand, the supplementation of a vitamin with different P-CPAs did not improve post-thaw motility except 2% GLY.

In the present study, the supplementation of saccharides with P-CPAs improved the ATP content than the control without the supplementation of saccharides. Particularly, sperm cryopreserved using 3% S + 8% DMSO showed improved ATP content than all types of cryopreserved sperm samples. Motility, MMP, and fertilization success depend on the ATP content of the sperm (Figueroa et al., 2017). ATP produced by mitochondria through oxidative phosphorylation is crucial for maintaining the suitable function of sperm during the fertilization process (Thuwanut et al., 2015; Figueroa et al., 2019). The possible explanation of the ATP content in sperm cryopreserved with CPAs supplemented with saccharides might be because saccharides can protect the sperm against membrane damage during the freeze-thaw process.

The sperm of Pacific abalone has an outer acrosome membrane, a plasma membrane, an outer mitochondrial membrane, and flagella. Thus, AI (vital parameter of fertility potential), PMI (crucial physiological indicator), and MMP (key indicator of mitochondrial activity) are quality indicators of sperm (Hossen et al., 2021b). In the present study, supplementation of saccharides with P-CPAs during cryopreservation improved AI, PMI, and MMP, hence improving post-thaw sperm quality. Similar findings have been reported for oyster (*C. angulate*) sperm (Anjos et al., 2021). Among various combinations, 3% S + 8% DMSO showed improved AI, PMI, and MMP of post-thaw sperm. Such improvements might be possible because saccharides can be used as energy sources. They can also reduce ice crystallization and decrease toxicity during the cryopreservation process (Tian et al., 2015; Hossen et al., 2021c). Saccharides have an osmotic shock protective role during extracellular water exhaustion caused by ice formation. They might also preserve the structural and functional integrity of cell membranes (Ohki et al., 2014).

DNA integrity is a crucial indicator of fertilization capacity and embryo quality (Shamsi et al., 2011; Liu et al., 2022). To provide secured genetic materials to the offspring, intact DNA is essential (Cabrita et al., 2010). The freeze-thaw process of cryopreservation may damage the post-thaw sperm DNA integrity (Hossen et al., 2021c). Rather than 2% G + 6% PG, other examined post-thaw samples showed significantly similar DNA integrity. However, in the present study, saccharides supplemented with P-CPAs improved the post-thaw sperm DNA integrity than those cryopreserved with P-CPAs alone (Hossen et al., 2021a). Present findings indicate that supplementing saccharides can improve the stability of post-thaw DNA integrity. Although saccharides cannot penetrate the plasma membrane, they can create an osmotic pressure and induce cell dehydration. It is known that saccharides can interact with plasma membrane phospholipids and increase sperm survivability during the freezing step of cryopreservation (Sarıözkan et al., 2012).

Quantitative RT-PCR (qPCR) is a vital technique to determine post-thaw sperm quality by assessing the mRNA content of sperm motility-regulating genes (Riesco et al., 2019; Hossen et al., 2021a). In marine mollusk, this method has been applied previously for abalone and oyster sperm (Riesco et al., 2019; Hossen et al., 2021a; Hossen et al., 2021b; Sukhan et al., 2022). It is well-known that the ion channel regulates the motility of sperm (Ren et al., 2001; Navarro et al., 2008). In this study, supplementing saccharides to the extender improved mRNA content of ion channel-regulating genes (HSP70, HSP90, and PKA-C) in post-thaw sperm than the control without the addition of saccharides (Hossen et al., 2021a). The same research group has previously reported such improvements when sperm were preserved using antifreeze protein supplemented with P-CPAs (Hossen et al., 2021b). However, post-thaw sperm had lower mRNA content of motility-associated ion channel-regulating genes than fresh sperm. A similar downregulation pattern of mRNA in cryopreserved sperm was previously reported in abalone and oyster sperm (Riesco et al., 2019; Hossen et al., 2021a; Hossen et al., 2021b). The present study also found that the mRNA content of motility-regulating genes (Axp66.0 and tektin-4) was downregulated in cryopreserved sperm than in fresh sperm. Sperm cryopreserved using 3% S + 8% DMSO showed higher mRNA content of Axp66.0 and tektin-4 than other examined samples. Downregulated mRNA content of tektin-4 was also previously reported in sperm cryopreserved using P-CPAs (Sukhan et al., 2022). The present study is the first to report the mRNA content of Axp66.0 in cryopreserved sperm of any organism. The possible explanation for such improvement in the mRNA content is that saccharides can protect sperm during cryopreservation. Present findings suggest that improved mRNA content of motility-regulating genes might be responsible for the improved motility of sperm cryopreserved using saccharides.

Furthermore, cryopreservation suppressed the mRNA content of fertilization protein (SP18-kDa). However, sperm cryopreserved using 3% S + 8% DMSO showed comparatively higher SP18-kDa mRNA content than sperm cryopreserved with other CPAs. The present study is the first to report the mRNA content of SP18-kDa in cryopreserved sperm in any organism. SP18-kDa is one of the principal acrosome proteins of abalone. SP18-kDa may bind a receptor gene of the egg plasma membrane and mediate egg-sperm fusion (Aagaard et al., 2010). This suggests that SP18-kDa might be used as a fertility marker of cryopreserved sperm.

Correlations among post-thaw sperm quality parameters showed that sperm DNA fragmentation was negatively correlated with other examined quality indicators. This finding suggests that reduced DNA fragmentation might be responsible for improved post-thaw sperm quality indicators. Negative correlations of DNA fragmentation with other quality indicators have been reported previously (Alcay et al., 2020; Hossen et al., 2021b).

Fertility and hatchability are vital indicators of the reproduction success of post-thaw sperm. Improved fertilization and hatching rates were observed during *in vitro* fertilization of abalone using saccharide (sucrose)-supplemented cryopreserved sperm compared to control sperm, cryopreserved without saccharides. Similar phenomena have been reported previously for cryopreserved greenlip abalone (Liu et al., 2014a) and salmon sperm (Sandoval-Vargas et al., 2021). Such improvement might be due to the protective effects of saccharides on sperm quality indicators of Pacific abalone as discussed in previous sections.

Previous studies have reported that sperm cryopreserved using an antifreeze protein (AFP) combined with 8% DMSO showed improved sperm quality than sperm cryopreserved using only 8% DMSO (Hossen et al., 2021a). The present finding also reported similar results, that is, 3% S combined with 8% DMSO also improved sperm quality than 8% DMSO only. Since sperm cryopreserved with an AFP or sucrose combined with 8% DMSO showed similar improvement in sperm quality, hatchery owners may use any combination of cryoprotectants for large-scale sperm cryopreservation of Pacific abalone and hatchery seed production. However, saccharides are heat-tolerant, cheaper, and more readily available than an AFP. Thus, they could be cost-effective cryoprotectants for the large-scale cryopreservation of abalone sperm.

## 5 Conclusion

The present study reports positive influences of saccharides on post-thaw sperm quality including motility, AI, PMI, MMP, ATP content, and the fertilization potential of sperm of Pacific abalone, *H. discus hannai*. Saccharides dramatically improved the DNA integrity of post-thaw sperm. Sperm cryopreserved using 3% S + 8% DMSO showed higher sperm quality and mRNA content, although the sperm showed insignificant differences in sperm quality indicators (except PMI) compared to those cryopreserved using 1% G + 8% EG. It could be concluded that 3% sucrose combined with 8% DMSO improved sperm quality compared to other examined cryoprotectants. All examined parameters including motility and fertility-associated gene expression proved that 3% sucrose combined with 8% DMSO for sperm cryopreservation was comparatively more suitable than other cryoprotectants for sperm cryopreservation and molecular conservation of Pacific abalone.

## Data Availability

The original contributions presented in the study are included in the article/Supplementary Materials; further inquiries can be directed to the corresponding author.
