# Supplementary material for: Saccharides Influence Sperm Quality and Expressions of Motility and Fertilization-Associated Genes in Cryopreserved Sperm of Pacific Abalone, Haliotis discus hannai
Source: Front Cell Dev Biol. 2022 Jul 19;10:935667. doi: 10.3389/fcell.2022.935667 (PMC9343956; doi:10.3389/fcell.2022.935667)
Supplement: Supplementary file 1 [file DataSheet1.docx]

**Influence of saccharides on** **cryopreserved sperm quality, motility and fertilization associated gene expression of Pacific abalone, *Haliotis discus hannai***


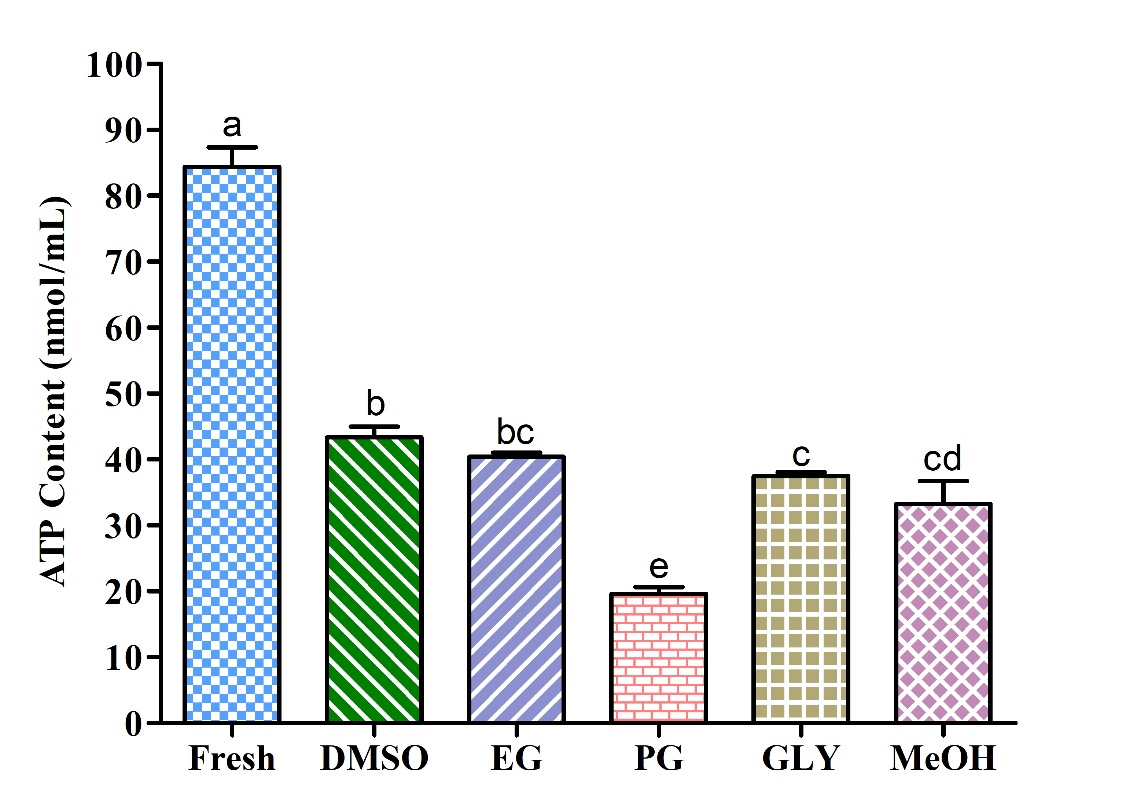


**Figure S1.** Adenosine triphosphate (ATP) content in different types of post-thaw sperm cryopreserved using P-CPA only.
